# The Moderating Role of Nonviolent Communication in the Relationship Between PTSD and Depressive Symptoms: A Longitudinal Investigation

**DOI:** 10.3390/healthcare13172163

**Published:** 2025-08-29

**Authors:** Chak Hei Ocean Huang, Hiu Lam Natasha Lau, Guangzhe Frank Yuan, Caimeng Liu, Stanley Kam Ki Lam, Hong Wang Fung

**Affiliations:** 1 Department of Social Work and Social Administration, The University of Hong Kong, Pok Fu Lam, Hong Kong; ocean9128@gmail.com; 2 Department of Psychology, University of Essex, Colchester CO4 3SQ, UK; natashalauu@gmail.com; 3 School of Education Science, Leshan Normal University, Leshan 614000, China; psyliu@lsnu.edu.cn; 4 Nethersole School of Nursing, The Chinese University of Hong Kong, Shatin, Hong Kong; stanleylam@cuhk.edu.hk; 5 School of Nursing, The Hong Kong Polytechnic University, Hung Hom, Hong Kong

**Keywords:** PTSD, depression, NVC, interpersonal relationships

## Abstract

**Background**: The demoralization model of depression suggests that PTSD could lead to depressive symptoms. However, not all people with PTSD have co-occurring depressive symptoms. **Objective**: To examine the moderating role of nonviolent communication (NVC) behaviors in the relationship between PTSD and depressive symptoms. **Methods**: Longitudinal data were obtained from a sample of young adults (*N* = 146) online. They completed validated screening measures of PTSD symptoms, depressive symptoms, and NVC behaviors at baseline (T1), and reported their depressive symptoms again after three months (T2). **Results**: PTSD symptoms were correlated to depressive symptoms at both T1 (*r* = 0.53, *p* < 0.001) and T2 (*r* = 0.37, *p* < 0.001). NVC behaviors were also negatively correlated to depressive symptoms at T1 (*r* = −0.40, *p* < 0.001) and T2 (*r* = −0.36, *p* < 0.001). After controlling for demographic variables, childhood trauma, and T1 depressive symptoms, T1 NVC behaviors moderated the effects of T1 PTSD symptoms on T2 depressive symptoms (*B* = 0.149, *p* = 0.019). T1 PTSD symptoms predicted T2 depressive symptoms only when the levels of NVC behaviors were low (β = −1.149, *p* = 0.001). **Conclusions**: The moderating role of NVC behaviors in the longitudinal relationship between PTSD and depressive symptoms was identified. NVC training programs might be beneficial to individuals with PTSD and depressive symptoms. Future studies are needed to evaluate the effectiveness of such programs for trauma survivors.

## 1. Introduction

Depression is a major public health concern worldwide, and it is also a leading cause of disability, contributing to a global burden of disease [1]. Depression is characterized by persistent sadness, loss of interest or pleasure in daily activities, and a variety of emotional and physical problems that can impair a person’s ability to function at work and home [2]. The high prevalence and considerable impacts of depression highlight the importance of further research on the etiology and treatment needs of depression [3].

While depression is a prevalent mental health problem and is categorized as a depressive disorder, it has been argued that depression is a heterogeneous condition with varying etiological factors, even if the clinical presentations may appear similar [4,5]. In other words, while two patients may present with similar depressive symptoms, their causes could be different, and thus they may require different intervention strategies. Rantala et al. [6] proposed that there could be different subtypes of depression, and some subtypes may be particularly related to psychological trauma and stress. It has been well-documented that people with post-traumatic stress disorder (PTSD) often suffer from co-occurring depression [7,8,9]. Based on the synchronous change model (i.e., PTSD and depression being time-synchronous with a possible third common factor) with a demoralization model (i.e., PTSD leading to depression) [10], some researchers have observed that PTSD symptoms could predict future depressive symptoms (e.g., [11,12]). Consequently, the experience of living with PTSD symptoms could be profoundly distressing and therefore lead to negative thoughts and emotions and avoiding behaviors, ultimately resulting in depression.

Nevertheless, the predictive role of PTSD symptoms on subsequent depression is not consistent across studies. For example, Schindel-Allon, Aderka, Shahar, Stein and Gilboa-Schechtman [10] found that PTSD symptoms do not always predict depressive symptoms. Therefore, in order to improve our understanding of the synchronous change model of trauma and depression, more studies are needed to explore what factors might affect or moderate the relationship between PTSD and depressive symptoms. Understanding the factors that influence the transition from PTSD to depression is crucial for developing effective interventions. However, little is known about what factors might moderate the longitudinal relationship between PTSD and depressive symptoms. Identifying potential moderating factors can provide insights into why some individuals with PTSD develop depression while others do not.

In this study, we proposed that communication behaviors, particularly nonviolent communication (NVC) behaviors, might be a potential moderator. NVC is a concept developed by Rosenberg and colleagues, referring to a communication approach that emphasizes (a) self-connection (e.g., self-empathy), (b) authentic self-expression, and (c) empathic listening [13,14]. The United Nations [15] has recognized the potential benefits of NVC in maintaining peace and resolving conflicts. NVC behaviors have been recently operationalized using a reliable and valid self-report measure [16]. It has also been observed that PTSD symptoms are associated with a lack of NVC behaviors [17]. As such, the aim of the present study is to investigate the moderating role of NVC behaviors in the relationship between PTSD symptoms and subsequent depressive symptoms. We hypothesized that the effects of PTSD symptoms on subsequent depressive symptoms would be stronger if the levels of NVC behaviors were low.

There are several reasons for proposing NVC as a potential moderator for the relationship between PTSD and depressive symptoms. First, individuals with trauma and PTSD are often characterized by self-blame and interpersonal difficulties [18]. Second, negative social–interpersonal experiences are significant predictors of negative outcomes in trauma survivors [19,20]. Third, negative social–interpersonal experiences also predict depressive symptoms [21,22]. Therefore, we hypothesized that PTSD would have stronger effects on depressive symptoms if the individuals exhibit fewer NVC behaviors, such as peacefully expressing themselves when communicating with others.

## 2. Materials and Methods

### 2.1. Participants

The longitudinal data of this study was collected through an international online survey project. Ethical approval was obtained from the institutional review board at Leshan Normal University, China. The study was conducted in accordance with the Declaration of Helsinki. The project aimed to investigate the psychosocial correlates of mental health among young adults. To increase the diversity of the sample, subjects were recruited through social media platforms such as Facebook and Instagram. With this survey project being an international one, online data collection was adopted under concerns for feasibility and cost-effectiveness [23]. Part of the baseline data has been reported elsewhere [24]. The inclusion criteria were as follows: (1) being 18 to 24 years old; (2) being able to comprehend English; and (3) providing online informed consent. The only exclusion criterion was self-reported clinical diagnosis of a reading disorder or intellectual disabilities.

A total of 283 participants met all inclusion criteria and provided valid responses at baseline (T1). After an average of 108.9 days (*SD* = 7.53), 146 participants provided valid responses at follow-up (T2) (retention rate = 53.7%). Data at both time points was matched using the unique email address of each participant. Participants from the following countries were included: Canada (25.4%), the United Kingdom (22.6%), Australia/New Zealand (21.2%), the United States (14.1%), and others (16.6%).

### 2.2. Measures

At T1, participants completed a demographic survey and self-reported measures of NVC behaviors, PTSD symptoms, depressive symptoms, and childhood trauma. Participants reported their depressive symptoms again at T2.

*Nonviolent Communication Behaviors Scale* (NVCBS). The NVCBS is a 7-item self-report measure which examines three specific types of NVC: self-connection (e.g., Knowing that my emotions stem from my own thoughts and unmet needs—my emotions are not caused by others), empathetic listening (e.g., Being open and flexible when communicating with others), and authentic self-expression (e.g., Willing to make requests for my own needs and remaining open to any response after requesting, not obsessed with a particular outcome) [16]. In the original validation study, it had exhibited satisfactory internal consistency (α = 0.79 to 0.81), positive correlation (*r* = 0.21) with empathy, and negative correlation (*r* = −0.20) with negative beliefs about emotions [16]. Items of this scale adopted a 4-point measurement (1 = never; 4 = frequently). Cronbach’s alpha for the NVCBS in the present sample was 0.70.

*PTSD Checklist for DSM-5* (PCL-5). The PCL-5 is a self-reported measure with 20 items that assesses the DSM-5 symptoms of PTSD. It was reported to have excellent internal consistency (α = 0.94), test–retest reliability (*r* = 0.82), and convergent validity (*r.s.* = 0.74 to 0.85) [25]. Since a 4-item version of the PCL-5 has also been proven to be psychometrically sound, accounting for 90.4% of the variance (*r* = 0.95) in the original scale [26], this abbreviated version was used in this study. Cronbach’s alpha for the abbreviated PCL-5 in the present sample was 0.83.

*Patient Health Questionnaire—Brief* (PHQ-B). The PHQ-B is the short version of the original PHQ-9 [27,28]. In subsequent studies, this version of the PHQ has exhibited good reliability and validity in capturing depressive symptoms [29,30]. Therefore, the PHQ-B, which consisted of Item 1, 2, and 9 of the PHQ-9, was used. Cronbach’s alpha for the PHQ-B in the present sample was 0.84 (T1) and 0.80 (T2).

*Brief Trauma Betrayal Survey (BBTS)*. The 12-item childhood trauma section of the BBTS was used to assess traumatic experiences during childhood. The BBTS has exhibited decent test–retest reliability for childhood trauma (83%) in an adult community sample [31]. It assesses 12 different types of childhood traumatic events.

### 2.3. Data Analysis

IBM SPSS Statistics 22.0 was used for statistical analyses. Descriptive statistics were used to present the sample characteristics. Correlation analyses were then conducted to examine the relationship that depressive symptoms had with other variables. We also conducted a hierarchical multiple regression and a moderation analysis using PROCESS V4.2 [32] to examine the longitudinal relationship between PTSD symptoms and T2 depressive symptoms, as well as the moderating effects of NVC behaviors, with bias-corrected 95% confidence intervals for 10,000 bootstrap iterations, after controlling for demographic variables (age, gender, education level, use of psychiatric services), childhood trauma and T1 depressive symptoms.

## 3. Results

### 3.1. Descriptive Statistics

This sample consists of 146 participants, with an average age of 20.1 (*SD* = 1.90). Of this sample, 92.5% were female, and 32.2% of them had used psychiatric services in the past 12 months. On the BBTS, 87.0% reported at least one childhood traumatic event. Sample characteristics are presented in Table 1. 

Among the variables, PTSD symptoms were found to have the highest correlation with depressive symptoms at both T1 (*r* = 0.53, *p* < 0.001) and T2 (*r* = 0.37, *p* < 0.001). Meanwhile, NVC behaviors were found to be the most negatively correlated with depressive symptoms at T1 (*r* = −0.40, *p* < 0.001) and T2 (*r* = −0.36, *p* < 0.001).

### 3.2. Moderation Analyses

To investigate the moderating effects of NVC behaviors, we first included PTSD symptoms and NVC behaviors in the model predicting T2 depressive symptoms, while controlling for demographic variables (age, gender, education level, use of psychiatric services), childhood trauma, and T1 depressive symptoms. Initially, neither PTSD symptoms nor NVC behaviors were significant predictors of T2 depressive symptoms. After that, we added the interaction between PTSD symptoms and NVC behaviors into the model. This produced a statistically significant result in the prediction model (Δ*R*^2^ = 0.046, *p* < 0.001), while also proving itself to be a significant predictor of T2 depressive symptoms (β = −1.149, *p* = 0.001) (see Table 2).

**Figure 1:**
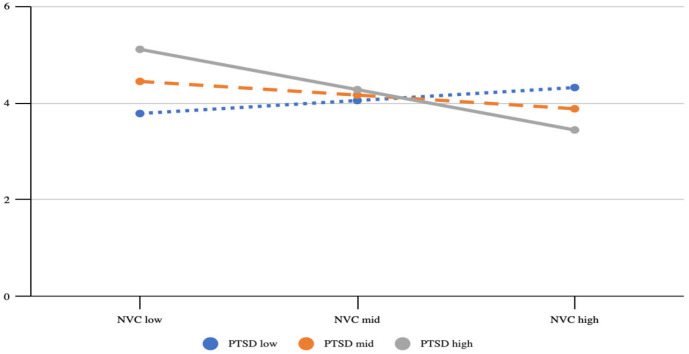
Conditional effects of PTSD symptoms on T2 depressive symptoms, with NVC behaviors as a moderator; covariates include demographic variables (age, gender, education level, use of psychiatric services), childhood trauma and T1 depressive symptoms. The plot was generated in SPSS PROCESS Model 1 to visualize the conditional effect of the focal predictor. The cutoffs of the independent variable and the moderator were generated from SPSS PROCESS Model 1 for probing the details of interaction and do not have implications on groupings. The Y axis was calibrated as the possible scores of the dependent variable (PHQ-B), which range from 0 to 9.

The subsequent interaction plot and Johnson–Neyman results also showed that the effect of PTSD symptoms on T2 depressive symptoms (*B* = 0.149, *p* = 0.019) was only statistically significant when the score of NVC behaviors was 15 or lower (see Table 3 and Figure 1).

The results indicate that NVC behaviors may buffer the effects of PTSD symptoms on subsequent depressive symptoms.

## 4. Discussion

This study aimed to explore the longitudinal relationship between PTSD and subsequent depressive symptoms, with a focus on the moderating effects of NVC behaviors. We found that PTSD symptoms predicted subsequent depressive symptoms only when the levels of NVC behaviors were low. This suggests that NVC behaviors act as a significant moderator that may reduce the relationship between subsequent depressive symptoms.

The above findings have provided valuable insights into understanding, prevention and treatment for people with comorbid PTSD and depressive symptoms. A moderator that could influence the relationship between PTSD and depressive symptoms was identified. NVC behaviors might help protect people with PTSD symptoms from developing depressive symptoms. The finding hence identified a potential moderator which could enrich the existing synchronous change model of trauma and depression [10,33]. Mental health practitioners such as social workers, psychologists and psychiatric nurses can work proactively with trauma survivors with PTSD symptoms to prevent the development of depressive symptoms. Moreover, such interventions could also be expanded to their family members, given their pivotal role in mental health recovery among traumatized and depressed individuals [34,35,36,37].

This study has several strengths: we employed a longitudinal design, included an international sample, utilized reliable and valid screening measures, and controlled for a variety of possible confounding variables. However, there are also some limitations. Firstly, our sample may not be representative enough due to the self-selection bias during recruitment. Most of the participants were female, as well. Secondly, while valid, measures for both PTSD and depressive symptoms may not be clinically comprehensive enough, as only shortened measures were used. Thirdly, the length of the follow-up period (i.e., 3 months) might be insufficient, alongside the relatively high attrition rate. Therefore, future studies should include a more representative sample and employ more comprehensive measures of PTSD and depression, so as to identify potential moderators and inform early interventions. Despite the limitations, this present study has highlighted the importance of NVC behaviors in preventing and treating depressive symptoms among trauma survivors.

## 5. Conclusions

In the present study, we revealed the potential protective effects of NVC behaviors, suggesting that NVC trainings or interventions may be helpful for trauma survivors with PTSD symptoms. It has been discussed that since intrapersonal conflicts and interpersonal difficulties are characteristics of traumatized individuals, communication skills training such as NVC interventions might be beneficial to this population [16,17]. Given the moderating role of NVC behaviors as identified in the present study, timely interventions promoting NVC behaviors might reduce the effects of PTSD symptoms on depressive symptoms. Future studies should develop NVC-based programs for trauma survivors and evaluate whether such interventions could serve as an alternative or adjunctive intervention for people with PTSD symptoms.

## Figures and Tables

**Table 1:** Sample characteristics and correlates of depressive symptoms at T1 and T2 (*N* = 146).

			Depressive Symptoms (*r*)	
Variables	*M* (*SD*)	*n* (%)	T1	T2
Age	20.1 (1.90)		−0.11	−0.20 *
Gender (female)		135 (92.5%)	−0.04	−0.02
Education (undergraduate or above)		28 (19.2%)	−0.08	−0.12
Seen a psychiatrist (past 12 months)		47 (32.2%)	0.25 ***	0.23 **
T1 PHQ-B	5.0 (2.82)		/	/
T2 PHQ-B	4.3 (2.60)		0.64 ***	/
T1 BBTS	3.5 (2.55)		0.27 ***	0.21 *
T1 PCL-5	8.5 (4.46)		0.53 ***	0.37 ***
T1 NVCBS	19.0 (3.55)		−0.40 ***	−0.36 ***

* *p* < 0.05 ** *p* < 0.01 *** *p* < 0.001.

**Table 2:** Hierarchical multiple regression predicting T2 depressive symptoms (*N* = 146).

Variables	β	*p*	*F*	*Adjusted R* ^2^	Δ*R*^2^	Δ*F*
Step 1			17.683 ***	0.408	0.433	17.683 ***
Age	−0.136	0.063				
Gender (female)	−0.001	0.988				
Education (undergraduate or above)	−0.004	0.955				
Seen a psychiatrist (past 12 months)	0.082	0.218				
T1 BBTS	0.052	0.441				
T1 PHQ-B	0.588	<0.001				
Step 2			14.671 ***	0.459	0.060	5.336 **
Age	−0.146	0.038				
Gender (female)	0.004	0.955				
Education (undergraduate or above)	0.012	0.860				
Seen a psychiatrist (past 12 months)	0.028	0.677				
Childhood trauma	0.032	0.646				
T1 PHQ-B	0.559	<0.001				
T1 PCL-5	1.177	0.001				
T1 NVCBS	0.297	0.033				
PTSD x NVC	−1.149	0.001				

** *p* < 0.01 *** *p* < 0.001.

**Table 3:** Conditional effects of the focal predictor (PTSD symptoms) on T2 depressive symptoms at values of the moderator (*N* = 146).

NVC Behaviors ^1^	*B*	*SE*	*t*	*p*	*LLCI*	*ULCI*
Low (15.453)	0.149	0.063	20.367	0.019	0.025	0.273
Mid (19.007)	0.025	0.047	0.534	0.595	−0.068	0.119
High (22.560)	−0.098	0.055	−1.798	0.075	−0.207	0.010

^1^ The cutoffs of the moderator were generated from SPSS PROCESS Model 1 for probing the details of interaction and they do not have implications on groupings.

## Data Availability

Data that support the findings of this study are available from the corresponding authors upon reasonable request.

## Abbreviations

The following abbreviations are used in this manuscript:

PTSD	Post-traumatic stress disorder
NVC	Nonviolent communication
NVCBS	NVC Behaviors Scale
PCL-5	PTSD Checklist for DSM-5
PHQ-B	Patient Health Questionnaire—Brief
BBTS	Brief Betrayal Trauma Survey
